# Optimizing Patient Record Linkage in a Master Patient Index Using Machine Learning: Algorithm Development and Validation

**DOI:** 10.2196/44331

**Published:** 2023-06-29

**Authors:** Walter Nelson, Nityan Khanna, Mohamed Ibrahim, Justin Fyfe, Maxwell Geiger, Keith Edwards, Jeremy Petch

**Affiliations:** 1 Centre for Data Science and Digital Health Hamilton Health Sciences Hamilton, ON Canada; 2 Department of Statistical Sciences University of Toronto Toronto, ON Canada; 3 Fyfe Software Hamilton, ON Canada; 4 Department of Biology University of Hawaii Hilo, HI United States; 5 Department of Computer Science University of Hawaii Hilo, HI United States; 6 Institute of Health Policy, Management and Evaluation University of Toronto Toronto, ON Canada; 7 Division of Cardiology Department of Medicine McMaster University Hamilton, ON Canada; 8 Population Health Research Institute McMaster University Hamilton, ON Canada

**Keywords:** medical record linkage, electronic health records, medical record systems, computerized, machine learning, quality of care, health care system, open-source software, Bayesian optimization, pilot, data linkage, master patient index, master index, record link, matching algorithm, FEBRL

## Abstract

**Background:**

To provide quality care, modern health care systems must match and link data about the same patient from multiple sources, a function often served by master patient index (MPI) software. Record linkage in the MPI is typically performed manually by health care providers, guided by automated matching algorithms. These matching algorithms must be configured in advance, such as by setting the weights of patient attributes, usually by someone with knowledge of both the matching algorithm and the patient population being served.

**Objective:**

We aimed to develop and evaluate a machine learning–based software tool, which automatically configures a patient matching algorithm by learning from pairs of patient records previously linked by humans already present in the database.

**Methods:**

We built a free and open-source software tool to optimize record linkage algorithm parameters based on historical record linkages. The tool uses Bayesian optimization to identify the set of configuration parameters that lead to optimal matching performance in a given patient population, by learning from prior record linkages by humans. The tool is written assuming only the existence of a minimal HTTP application programming interface (API), and so is agnostic to the choice of MPI software, record linkage algorithm, and patient population. As a proof of concept, we integrated our tool with SantéMPI, an open-source MPI. We validated the tool using several synthetic patient populations in SantéMPI by comparing the performance of the optimized configuration in held-out data to SantéMPI’s default matching configuration using sensitivity and specificity.

**Results:**

The machine learning–optimized configurations correctly detect over 90% of true record linkages as definite matches in all data sets, with 100% specificity and positive predictive value in all data sets, whereas the baseline detects none. In the largest data set examined, the baseline matching configuration detects possible record linkages with a sensitivity of 90.2% (95% CI 88.4%-92.0%) and specificity of 100%. By comparison, the machine learning–optimized matching configuration attains a sensitivity of 100%, with a decreased specificity of 95.9% (95% CI 95.9%-96.0%). We report significant gains in sensitivity in all data sets examined, at the cost of only marginally decreased specificity. The configuration optimization tool, data, and data set generator have been made freely available.

**Conclusions:**

Our machine learning software tool can be used to significantly improve the performance of existing record linkage algorithms, without knowledge of the algorithm being used or specific details of the patient population being served.

## Introduction

The World Health Organization [1] has identified electronic patient record management as an essential part of modern health care. Delivering quality care and maintaining patient safety requires that the patient record available at the point of care is reflective of the full patient history. In high-income countries, patient record fragmentation can result in medical errors, and linkage has a high cost [2,3]. The problem is particularly challenging in low- and middle-income countries, where patients do not necessarily have a unique identifier, such as Myanmar [4-6]. This challenge necessitates probabilistic record linkage, where a health care provider is given the opportunity to link multiple records from the same patient in a master index [7].

In these settings, computer-assisted patient record linkage has been shown to be effective for reconciling patient records from multiple sources [8]. Record linkage approaches can be divided into 2 categories: deterministic, where a fixed, unique identifier is used to join new pairs of records, or probabilistic, where a combination of patient attributes, such as name, location, and date of birth are used to score possible links, and linkage decisions are made based on these scores [8,9].

SantéMPI (SanteSuite Inc) is an open-source master patient index that has been deployed at scale in several low- and middle-income countries. SantéMPI integrates with several existing electronic health record solutions and supports all requirements defined by the Open Health Information Exchange, such as on the web or off the web capability, HL7 standards support, and mobile registration. SantéMPI implements a modern, validated hybrid record linkage approach in 2 stages [8]. In the first stage, the blocking stage, the set of all possible pairs in the database is filtered to a more tractable subset of possible pairs. For example, this might include ensuring that both records in the candidate pair have a patient’s date of birth in the same year. In the second stage, the scoring stage, each of the filtered candidate pairs is scored according to any number of attributes, such as name or address similarity, or whether the 2 records record the same gender. This scoring depends on a number of numeric parameters, such as how strongly to weight a given patient attribute [8,10].

The choice of how strongly to weigh a given patient attribute in the match-scoring stage depends on both the technical details of the matching algorithm used and the patient population under consideration. For example, in locales where the surname distribution is heavily skewed to a handful of surnames, it is less useful to match on surnames; likewise for matching on home addresses in regions where addresses do not have a standard form. It can be difficult to know in advance what attributes will be useful for matching patient records to one another for a given jurisdiction. While the record linkage approach attempts to provide a matching configuration with a reasonable set of default configuration options, human intervention is often required to curate patient record links.

In machine learning, numerous methods have been developed for optimizing the parameters of algorithms in ways that are agnostic to the implementation details of those algorithms. These techniques are known as black-box optimization and are widely applied to industrial optimization problems, hyperparameter tuning in deep learning, and drug delivery [11-13]. Bayesian optimization (BO), the black-box optimization algorithm used in this study, has been applied to privacy-preserving record linkage problems previously. In particular, Yu et al [14] showed that BO can successfully optimize the hyperparameters of a privacy-preserving record linkage algorithm by means of heuristics that are available even when ground-truth record linkages and nonlinkages are not.

In this paper, we present a novel application of black-box optimization to the problem of patient record linkage when ground-truth linkages and nonlinkages are available. Unlike previous work, we do not propose a new record linkage algorithm. Instead, we seek to build on an existing record linkage algorithm and propose to use BO to optimize the parameters of that algorithm using ground-truth linkages and nonlinkages. In this way, our approach is agnostic to the choice of the underlying record linkage algorithm and is easily adapted to other settings. Our approach is validated by integrating with SantéMPI, using the BO procedure to select the optimal patient attribute weights for record linkage.

## Methods

### Data Acquisition and Synthesis

We evaluated our approach using the 4 synthetic data sets distributed with the Freely Extensible Biomedical Record Linkage (FEBRL) system, along with an additional custom data set generated using FEBRL’s data set generation tool [15]. The 4 synthetic data sets contain varying numbers of patients, matches, and nonmatches, and were designed specifically for assessing new record linkage approaches. We also sought to evaluate the SantéMPI matcher and our configuration optimization approach on data with characteristics not typical of Western patient databases. In Hawaii, a majority of the population identifies as Asian, Native American, Pacific Islander, or 2 or more races [16]. The phonetics and spelling of Native Hawaiian names are also distinct. For example, due to the Native Hawaiian alphabet containing only 13 characters, vowel repetition is common [17]. In addition, the ’okina character (often represented in the Latin alphabet with an apostrophe), which is common in Native Hawaiian names, is not supported for many types of government records [18]. Publicly available data sources reflective of the population of Hawaii were obtained, and these sources were used as input to FEBRL’s data set generation tool. We briefly summarize the FEBRL data set generation process. First, the data sources (eg, names and addresses) are randomly sampled to generate “original” records. Second, the original records are mutated at random (possibly more than once) to create 1 or more “duplicate” records. The goal is to imitate common errors (such as data entry errors) that master patient indices such as the SantéMPI aim to resolve.

Characteristics of the data sets are provided in Table 1. The synthetic data distributed with FEBRL are accessible in the freely available Record Linkage Toolkit Python package [19].

**Table 1:** Description of the data used for evaluating the configuration optimization approach.

Data set	Description	Original records, n	Duplicates, n
FEBRL1^a^	Distributed with the FEBRL package.	500	500 (1 per original)
FEBRL2	Distributed with the FEBRL package.	4000	1000 (maximum 5 per original)
FEBRL3	Distributed with the FEBRL package.	2000	3000 (maximum 5 per original)
FEBRL4	Distributed with the FEBRL package.	5000	5000 (1 per original)
Hawaii	Constructed with the FEBRL data set generator using a number of Hawaii-specific data sources.	1000	1000 (maximum 5 per original)

^a^FEBRL: Freely Extensible Biomedical Record Linkage.

### Machine Learning Approach

We use an existing implementation of the BO algorithm, a black-box optimization technique, to identify the optimal parameters of the probabilistic scoring stage of the patient matching algorithm [20]. BO is an iterative procedure that optimizes a function, often used when the function is expensive to evaluate. In our framework, we use BO to identify the set of inputs (patient attribute weights in the matching configuration) that maximizes our objective (area under the receiver operator characteristic of the matching algorithm with the given configuration, as evaluated in historical linkages and nonlinkages). BO first randomly selects a set of configuration options, performs the matching with this configuration, and evaluates the matching performance according to the selected target metric, area under the receiver operating characteristic curve (AUROC). BO then modifies the configuration, performs matching once again, and evaluates the configuration using AUROC, updating its information about the optimal configuration. It selects the next configuration to maximize the acquisition function, which we choose to be expected improvement [21]. The modeling approach underlying BO is a Gaussian process, which is a nonparametric Bayesian regression technique, requiring the specification of a kernel. We use the default kernel in the BayesianOptimization library, the Matern kernel with smoothness parameter *ν*=2.5, which specifies that the function mapping configuration parameters to AUROC will be approximated by a twice-differentiable function. The length scale parameter of the kernel is learned during the optimization process [22].

The BO procedure optimizes the correlation between the matching scores and ground-truth matching labels, but it does not provide a way to select the matching score threshold for defining a match or nonmatch. Therefore, after the BO procedure completes, the configuration optimization routine sets the decision threshold for definite matches such that a minimum specificity of 100% is maintained in the training set (to minimize the risk that low-confidence record pairs are matched without human intervention). The threshold for possible matches is set to maximize sensitivity while keeping the fraction of record pairs necessitating human review under a user-specified threshold. For all evaluations, we set the human review threshold using the fraction of record pairs requiring review using the baseline configuration in the training set.

The BO and threshold optimization routines are implemented in Python 3 [23], using the BayesianOptimization [24], NumPy [25], and scikit-learn [26] libraries.

### Application Programming Interface Design and SantéMPI Integration

The BO tool communicates with the patient index via an HTTP API. Table 2 briefly describes the functionality of each HTTP end point. The general nature of the API ensures that the configuration optimization tool can be applied to any patient data storage application, simply by writing an integration layer.

As a proof of concept for the purpose evaluation, we integrated our configuration optimization tool with SantéMPI. SantéMPI does not implement this API natively, so a custom integration layer was written, which translates each API call to a SantéMPI-specific API call. This integration layer has been open-sourced as part of the SanteSuite project. The integration layer communicates ground-truth linkages using a Fast Healthcare Interoperability Resources API [27] and is therefore compatible with any Fast Healthcare Interoperability Resources–compatible clinical data repository software out of the box, provided the software exposes the additional endpoints for reading and updating the matching configuration.

**Table 2:** HTTP API^a^ end points that are required for the configuration optimization tool. All payloads and responses are formatted in JSON.

End point	Functionality	Payload	Response
GET /matchConfig/:configId/spec	Provide the specification of the configuration	—^b^	A dictionary with root key “attributes,” which is a dictionary mapping each configuration parameter to a dictionary with a key “bounds” with its valid lower and upper bounds on its range.
GET /matchConfig/:configId	Get the current configuration	—	A dictionary with root key “attributes,” which is a dictionary mapping each configuration parameter to its float value.
PUT /matchConfig/:configId	Set a new configuration	A dictionary with root key “attributes,” which is a dictionary mapping each configuration parameter to its new value.	—
GET /matchConfig/:configId/$groundTruthScores	Get the matching scores for a configuration	—	A dictionary with 2 keys, “0” and “1.” “0” maps to a list of matching scores for record pairs that were deemed nonmatches by a human, and “1” maps to a list of matching scores for record pairs that were deemed matches by a human.

^a^API: application programming interface.

^b^No payload or response.

### Evaluation

We split each data set at random into a training set (80% of patients, original records, and their duplicates) and evaluation set (the remaining 20% of patients). In the training set, all known matches were used as confirmed links. We randomly sampled a subset of known nonmatches to be used as confirmed nonlinks, in order to mimic how the tool would be used in practice. Using the default matching configuration as the initialization point for the BO procedure, we run the BO and threshold optimization procedures in a database containing the 80% training data.

SantéMPI reports linkages with 2 decision thresholds. High-confidence (“definite”) matches can be matched automatically without further human intervention. Lower-confidence (“possible”) matches must be reviewed manually prior to matching. To evaluate the performance of a given configuration, the sensitivity, specificity, and positive predictive value (PPV) of the matching algorithm were assessed using ground-truth labels in the evaluation set for both possible and definite matches. These metrics were also computed for the default matching algorithm configuration distributed with SantéMPI as a baseline, which implements the probabilistic matching algorithm described in reference [8]. We construct CIs for these metrics via bootstrapping and report these CIs [28].

### Ethical Considerations

We confirm that this research involved no human subjects, and all data used were artificially synthesized using the FEBRL software or distributed with the FEBRL software package.

## Results

For all data sets, the baseline configuration fails to detect any definite matches, corresponding to a sensitivity of 0%, a specificity of 100%, and an undefined PPV. The optimized configurations substantially improve the sensitivity in all data sets (Table 3), with no decrease in specificity and a PPV of 100%.

In addition, we report the sensitivity and specificity for matches predicted as possible or definite (Table 4). In all cases, the machine learning procedure results in a configuration with a significant improvement in sensitivity, at the expense of a modest decrease in specificity.

**Table 3:** Performance comparison of the baseline and machine learning–optimized matching configurations in SantéMPI in the held-out evaluation sets, for the detection of definite linkages not needing manual review.

Data set	Sensitivity (%; 95% CI)	Patients, n	Correctly predicted linkages (ground-truth linkages)
FEBRL1^a^	98.0 (95.0-100.0)	100	98 (100)
FEBRL2	96.6 (93.9-98.6)	800	196 (203)
FEBRL3	94.9 (93.0-96.5)	400	558 (588)
FEBRL4	98.3 (97.5-99.1)	1000	983 (1000)
Hawaii	96.6 (93.7-98.9)	200	168 (174)

^a^FEBRL: Freely Extensible Biomedical Record Linkage.

**Table 4:** Performance comparison of the baseline and machine learning–optimized matching configurations in SantéMPI in the held-out evaluation sets, for detection of possible record linkages needing manual review.

Data set	Sensitivity (%)			Specificity (%)			Positive predictive value (%)	
	Baseline (95% CI)	Optimized (change; 95% CI)	Baseline (95% CI)		Optimized (change; 95% CI)	Baseline (95% CI)		Optimized (change; 95% CI)
FEBRL1^a^	95.0 (90.1 to 98.9)	100.0 (+5.0%; 1.1 to 9.6)	100.0 (100.0 to 100.0)		99.4 (−0.6%; −0.4 to −0.7)	100.0 (100.0 to 100.0)		62.9 (−37.1%; −44.4 to −29.8)
FEBRL2	88.7 (84.3 to 92.8)	100.0 (+11.3%; 7.0 to 16.0)	100.0 (100.0 to 100.0)		99.3 (−0.7%; −0.6 to −0.7)	100.0 (100.0 to 100.0)		15.7 (−84.3%; −86.3 to −82.3)
FEBRL3	87.8 (85.1 to 90.3)	100.0 (+12.2%; 9.9 to 15.0)	100.0 (100.0 to 100.0)		99.3 (−0.7%; −0.7 to −0.7)	98.9 (97.9 to 99.6)		26.6 (−72.3%; −74.3 to −70.3)
FEBRL4	90.2 (88.4 to 92.0)	100.0 (+9.8%; 8.0 to 11.6)	100.0 (100.0 to 100.0)		95.9 (−4.1%; −4.1 to −4.0)	100 (100.0 to 100.0)		2.4 (−97.6%; −97.7 to −97.5)
Hawaii	93.1 (88.9 to 96.6)	100.0 (+6.9%; 3.4 to 11.1)	100.0 (100.0 to 100.0)		98.9 (−1.1%; −1.2 to −0.9)	99.4 (97.9 to 100.0)		31.9 (−67.5%; 71.6 to −63.6)

^a^FEBRL: Freely Extensible Biomedical Record Linkage.

## Discussion

### Principal Findings

Our results show that BO is a viable technique for improving the performance of probabilistic record linkage in a clinical context. In data sets designed for the validation of new record linkage approaches, our configuration optimization tool successfully identifies patient attribute weights that offer significantly improved performance according to sensitivity and AUROC (Tables 3 and 4, and Table S1 in Multimedia Appendix 1), with only a modest decrease in specificity. In addition, by validating in a synthetic Hawaiian population with linguistic characteristics that differ from commonly used Western data sets, we have shown that the approach may be promising in jurisdictions that do not use the standard English alphabet.

Our procedure has 2 stages: in the first, BO optimizes the matching algorithm parameters to maximize the correlation between known ground-truth linkages and nonlinkages and predicted match scores. In the second stage, we must select match score decision thresholds to report (in the case of SantéMPI) possible and definite matches and nonmatches to the user. The statistically significant improvement in AUROC (Table S1 in Multimedia Appendix 1) for the 4 largest data sets confirms that the BO procedure improves the concordance of match scores with ground-truth labels and that it is at least in part the BO procedure, which confers the statistically significant gains in sensitivity reported across all 5 data sets (Tables 3 and 4) and not the decision threshold selection.

In record linkage, as in all classification problems, one must trade-off between the cost of false positives and false negatives when selecting the decision threshold. In the evaluation presented in this study, 2 decision thresholds determine whether a record pair is linked by the system without further human intervention (definite matches), presented for human review (possible matches), or never presented for human review. This differs from record linkage in a research context, where most commonly a record pair will only ever be linked or not linked [29,30]. Due to this additional complexity, we establish the value of the machine learning optimization procedure in 2 stages, evaluating both definite and possible match predictions.

When identifying definite matches, a false positive prediction has a high cost, because the records of 2 distinct patients will be incorrectly linked, leading to potential privacy issues, increasing the risk of medical errors, and reducing the credibility of patient records [31]. A false negative prediction has a comparatively lower cost because that record pair may still be reviewed by a human. We show that the baseline matching configuration fails to identify any definite linkages in all data sets examined, due to the fact that the definite match decision threshold is set too conservatively by default. In contrast, the machine learning–optimized configuration selects a decision threshold that correctly identifies the vast majority of true linkages, with no decrease in specificity. This represents a substantial reduction in the human review effort required to identify the vast majority of record linkages (Table 3) because these linkages will be made without any additional human review. By maintaining a specificity of 100% across all data sets considered when identifying definite matches, we have shown that this decrease in human review burden does not come at the expense of false positives, which have the aforementioned high cost.

When identifying possible matches, a false positive prediction confers additional human review burden, while a false negative prediction corresponds to a patient record that will remain fragmented. Like incorrect linkages, a fragmented patient record has a high cost. We show that the baseline matching configuration has less-than-perfect sensitivity for identifying possible matches, leaving up to 9.8% of patient records fragmented, never presenting them for human review. In contrast, the machine learning–optimized configuration identifies all ground-truth linkages as possible matches in all data sets, though in this case at the cost of decreased specificity, representing an increase in the amount of human review to identify these more difficult linkages (Table 4).

It is important to interpret the results of Tables 3 and 4 jointly. For example, with the baseline configuration applied to FEBRL1, 95 record pairs would need to be manually reviewed to recover the first 95 matches, and the remaining 5 record pairs would never be presented for human review (missed linkages). In contrast, after optimization, 98 record pairs would be matched automatically, while fewer than 5 records would need to be manually reviewed to flag the remaining 2 matches (resulting in no missed linkages with minimal additional human review, despite the apparently large drop in PPV, and no false linkages). This is due to the choice of maximizing the sensitivity in the detection of possible linkages. We emphasize that this choice of maximizing sensitivity at the expense of human review burden is unrealistic in some scenarios, best exemplified in our tests by the FEBRL4 data set. The use of data-driven decision threshold selection in this evaluation does not preclude manual intervention in deployment, for example, by selecting the trade-off between the human review required for possible match predictions and correctly identifying all linkages. Indeed, because human review is not without error, and the cost of a false linkage is high, in many settings it may be useful to sacrifice perfect sensitivity in detecting possible linkages for better specificity by manually tuning the decision threshold. The proposed software tool provides performance metrics to the user, detailing these trade-offs and allowing them to make informed decisions. In an effort to make our findings reproducible, we do not present results that are the result of manual tuning of decision thresholds.

### Limitations

The primary limitation of our study is the use of synthetic data, which may not accurately reflect the way our tool would be used in practice. We mitigated this by consulting experts in global digital health to ensure that the synthetic data were as realistic as possible, and by making our tool freely available and open-source, we have reduced the overhead of future evaluation in real-world patient populations and settings. Additionally, because the worst-case performance of our approach automatically falls back to the default implementation, the risk of deployment based on the results in synthetic populations is minimal. In particular, in our implementation, the BO routine directly optimizes the AUROC, and so will never return an optimized configuration with worse performance than the initial configuration according to AUROC. Finally, the tool reports detailed evaluation metrics to the user and allows them to set custom decision thresholds, but doing so requires domain knowledge.

### Conclusions

Effective patient record linkage is critical in the deployment of patient record management software. A given patient-matching algorithm should be adapted to the population being served. BO, as implemented in our freely available, open-source tool, provides a means to automatically adapt a patient-matching algorithm to a new population.

## Appendix

Multimedia Appendix 1 Additional metrics and information for reproducing the analyses.

## Abbreviations

API: application programming interface
AUROC: area under the receiver operating characteristic curve
BO: Bayesian optimization
FEBRL: Freely Extensible Biomedical Record Linkage
MPI: master patient index
PPV: positive predictive value

## Data Availability

All data used in this study are freely available as part of the existing Record Linkage Toolkit Python package, and the Hawaii data sources are made available at links available in Multimedia Appendix 1. All software used for our implementation, including SantéMPI, the middleware, and the configuration optimization tool are made available at GitHub.
