# Supplementary material for: Optimizing Patient Record Linkage in a Master Patient Index Using Machine Learning: Algorithm Development and Validation
Source: JMIR Form Res. 2023 Jun 29;7:e44331. doi: 10.2196/44331 (PMC10365597; doi:10.2196/44331)
Supplement: Multimedia Appendix 1 [file formative_v7i1e44331_app1.docx]

# Multimedia Appendix 1

## Additional Performance Metrics

We also assessed the ranking performance (AUROC) in each of the held-out evaluation sets. In all cases, the machine learning procedure improves the ranking performance of the underlying matching algorithm. This improvement was statistically significant in all but the smallest dataset, FEBRL1.

| **Table S1.** Improvement in ranking performance for the machine learning-optimized configuration over the baseline configuration, as measured by the change in area under the receiver operator characteristic (AUROC). | |
| --- | --- |
| **Dataset** | **Improvement in AUROC (95% CI)** |
| FEBRL1 | +9.4 x 10^-5^ (-2 x 10^-6^, 2.3 x 10^-4^) |
| FEBRL2 | +8.7 x 10^-5^ (2.2 x 10^-5^, 1.7 x 10^-4^) |
| FEBRL3 | +3.2 x 10^-4^ (1.1 x 10^-4^, 5.7 x 10^-4^) |
| FEBRL4 | +2.2 x 10^-4^ (1.1 x 10^-4^, 3.8 x 10^-4^) |
| Hawaii | +6.9 x 10^-6^ (5.1 x 10^-7^, 1.7 x 10^-5^) |

*Note that AUROC can be interpreted as the empirical probability that a known linkage has a higher match score than a known non-linkage. These estimates are therefore small due to the denominator being the squared total number of record pairs.*

## SanteMPI Evaluation

In this section, we aim to provide enough detail to reproduce our analyses fully, enabled by the fact that all software in this study is open-source. As described in the Methods section, all data used in this study is freely available as part of the existing Record Linkage Toolkit Python package, and the Hawaii data sources are made available at [https://github.com/mgeiger00/febrl_hawaii](https://github.com/mgeiger00/febrl_hawaiizf). SanteMPI, the middleware, and the configuration optimization tool are made available at <https://github.com/santedb>.

For each dataset, we used the following procedure to obtain the reported performance estimates:

1. Randomly split the dataset’s “original” records into 80% train, and 20% test. Construct the training and test sets using the original records with their corresponding known duplicates.
2. Manually seed the training set into a SanteMPI SQL database, and the test set into another database.
3. Set the match configuration to the default (baseline). Run the configuration optimization tool in read-only mode while pointed at the test database to obtain the baseline match scores and labels for the test set.
4. Optimize the match configuration while pointed at the training database by running the configuration optimization tool for 50 iterations.
5. Run the configuration optimization tool in read-only mode while pointed at the test database to obtain the machine learning-optimized match scores and labels for the test set.
6. Evaluate AUROC, sensitivity, specificity, and positive and negative predictive values in the baseline and machine learning-optimized match scores, using the ground-truth labels in the test set.
